# Elder abuse in Norwegian nursing homes: a cross-sectional exploratory study

**DOI:** 10.1186/s12913-019-4861-z

**Published:** 2020-01-03

**Authors:** Anja Botngård, Arne Henning Eide, Laura Mosqueda, Wenche Malmedal

**Affiliations:** 1 Department of Public Health and Nursing, Norwegian University of Science and Technology, Trondheim, Norway; 2 Department of Health Research, SINTEF Digital, Oslo, Norway; 3 Keck School of Medicine of University of Southern California, Los Angeles, USA

**Keywords:** Elder abuse, Elder mistreatment, Nursing homes, Primary care, Nursing staff, Perpetrated abuse, Observed abuse

## Abstract

**Background:**

Elder abuse is a global public health and human rights problem that is predicted to increase as many countries experience a rapid growth in their population of older adults. Elder abuse undermines an older person’s well-being and is associated with a range of serious health consequences. In institutional care settings, older residents are particularly vulnerable and hence at higher risk of being abused, but few countries have explored the extent and nature of this phenomenon in national studies. The aim of this study is to estimate the prevalence of observed and perpetrated staff-to-resident abuse in Norwegian nursing homes.

**Methods:**

We conducted a cross-sectional exploratory study of nursing staff in 100 randomly drawn Norwegian nursing homes. Nursing staff completed a pen and paper survey measuring how often during the past year they had observed staff commit acts of neglect and psychological, physical, financial/material, and sexual abuse towards residents. They also reported how often they had perpetrated acts of abuse themselves, and these rates were disaggregated by nursing staff’s gender, age and education.

**Results:**

Of 3693 nursing staff (response rate 60.1%), 76% had observed one or more incidents of elder abuse during the past year, and 60.3% reported they had perpetrated one or more incidents of abuse in the same period. Psychological abuse and neglect were most commonly reported. Male staff reported more acts of physical abuse, while female staff reported more acts of neglect. Higher education of staff was associated with higher rates of self-reported psychological abuse, physical abuse and neglect.

**Conclusions:**

This first national survey of staff in Norwegian nursing homes is one of the largest studies globally estimating the prevalence of elder abuse in institutional settings. Overall, we found staff-to-resident abuse to be relatively common, and our findings propose a need for preventive strategies to improve the quality of life and safety of residents in Norwegian nursing homes.

## Background

Elder abuse is a global public health and human rights problem, and the mistreatment of older people is associated with a range of negative health outcomes from minor injuries to lasting disabilities, long-term psychological problems, suicide attempts, and increased risk of hospitalization, institutionalization and premature death [1–6]. Moreover, elder abuse is related to societal consequences such as medical costs of emergency care, hospitalization, and expenses linked to the prosecution, punishment and rehabilitation of perpetrators [4, 7, 8]. The Centers for Disease Control and Prevention (CDC) defines elder abuse or mistreatment as *“an intentional act or failure to act by a caregiver or another person in a relationship involving an expectation of trust that causes or creates a risk of harm to an older adult* [9]. This includes psychological, physical, financial/material and sexual abuse, and intentional or unintentional neglect.

Compared with research on intimate partner and sexual violence, little has been done to shed light on the mistreatment of older adults [10]. Moreover, the majority of elder abuse studies have been conducted in the community and not in institutional settings [11, 12], where residents tend to be more frail and vulnerable to abuse [13]. In 2017, the first meta-analysis on the global prevalence of elder abuse in both community and institutional settings estimated a pooled prevalence of 10.0% (CI 95, 5.2–18.6%) when reported by older adults themselves, and 34.3% (CI 95, 22.9–47.8%) when reported by caregivers or third parties [12]. In 2019, another systematic review and meta-analysis estimated the prevalence of elder abuse in institutional settings and found that 64.2% (CI 95, 53.3–73.9%) of staff admitted perpetrating at least one incident of abuse during the past year [14]. Among the subtypes of abuse, the prevalence of staff-reported psychological abuse was 32.5%; neglect 12.0%; physical abuse 9.3%, and sexual abuse 0.7%, and these rates were even higher when reported by older residents themselves [14].

Existing literature does however provide a wide range of prevalence estimates, influenced by the perspective from which the abuse is measured and understood, definitions and data collection methods used, and variation in reference periods to measure the extent of abuse [12, 14–20]. A literature synthesis found approximately 40 definitions and several subtypes of abuse [20]. For example, where some defined verbal and medication abuse as unique categories [21–23], others included acts of verbal character under psychological abuse, and misuse of medications as neglect or physical abuse [9, 24]. Different data collection methods are also a significant cause of the variability in estimates, where most measurement instruments are self-designed and study-specific [12]. The use of different reference periods might also impact the prevalence, where some studies use a four week period [25], while others use three months [21–23] or even the entire work career [26]. Nevertheless, a past-year reference period is the most commonly used [24, 27–33].

Elder abuse is a complex interplay of individual, relationship, social and cultural factors, and “risk factors” rather than “causes” is more commonly used in the study of elder abuse [34, 35]. Bronfenbrenner’s ecological model was introduced to the field of violence in the late 1970s, and in 2011, Schiamberg et al. [36] applied this model to illustrate the distinctive risk factors of elder abuse in nursing homes. This model comprises five levels, where the first level (micro) focuses on individual characteristics such as biological and demographic factors that increase the likelihood of being a victim or perpetrator of abuse. The second level (meso) explores how social relationships between residents and staff increase the risk of victimization and perpetration of abuse. The third level (exo) examines institutional factors in which these relationships are embedded, and the fourth level (macro) explores larger societal factors such as cultural norms, ageism/sexism, and public policy/economy. The fifth and final level seeks to identify changes in the environment over time [34–37].

Few studies have been conducted on risk factors of elder abuse in institutional care settings [37], and existing research is ambiguous when describing the individual-level risk factors of staff. For instance, in Irish nursing homes, male staff reported committing more acts of neglect than their female colleagues [24], and in Swiss nursing homes, men admitted more acts of emotional abuse [25]. In Taiwan, younger staff committed more psychological abuse [38], and in Norway, older staff reported more acts of physical abuse [26]. The Norwegian study also found that higher-educated staff admitted perpetrating more acts of physical and psychological abuse, in contrast to Israel, where nurse aides and practical nurses admitted to more acts of mental abuse compared to registered nurses [30].

While international research agrees on the persistent occurrence of elder abuse and its devastating consequences, the World Health Organization’s (WHO) *Global status report on violence prevention 2014* [10] emphasized that elder abuse was less addressed in governmental action plans than the other forms of interpersonal violence. The Norwegian government has also, in many strategic white papers and national action plans, highlighted elder abuse as a societal problem. Still, the first national study on violence and abuse reported by community-dwelling older adults aged 65 and over was conducted in 2017, where the overall prevalence rates were estimated to be between 6.8 and 9.2% [39].

The Norwegian population above 80 years of age will more than double by the year 2060 [40], and at the same time, it is predicted that health care services will have a substantial staff shortage [41]. This combination of exponential growth in the number of older adults and an inadequate supply of trained nursing staff is dangerous, and could lead to a deterioration of health services for residents in Norwegian nursing homes [42]. The completion of this research establishes a baseline on the magnitude of the problem, so appropriate interventions to reduce or prevent elder mistreatment can be developed, implemented and evaluated. The primary objectives of our study were to 1) estimate the prevalence of observed and perpetrated staff-to-resident abuse in Norwegian nursing homes and 2) explore demographic differences between staff who reported perpetrating and not-perpetrating acts of abuse.

## Methods

### Study design

We conducted a cross-sectional exploratory pen and paper survey of nursing staff in Norwegian nursing homes during October 2018 and January 2019.

### Setting

All public and private nursing homes or retirement homes, hereafter called nursing homes, registered in the Central Register of Establishments and Enterprises (CRE), were eligible for inclusion. In Norway, municipalities own and operate approximately 90% of nursing homes, and private for-profit agencies or non-profit organizations typically set up as foundations operate about 10% [43].

### Randomization and recruitment of nursing homes

**Fig. 1:**
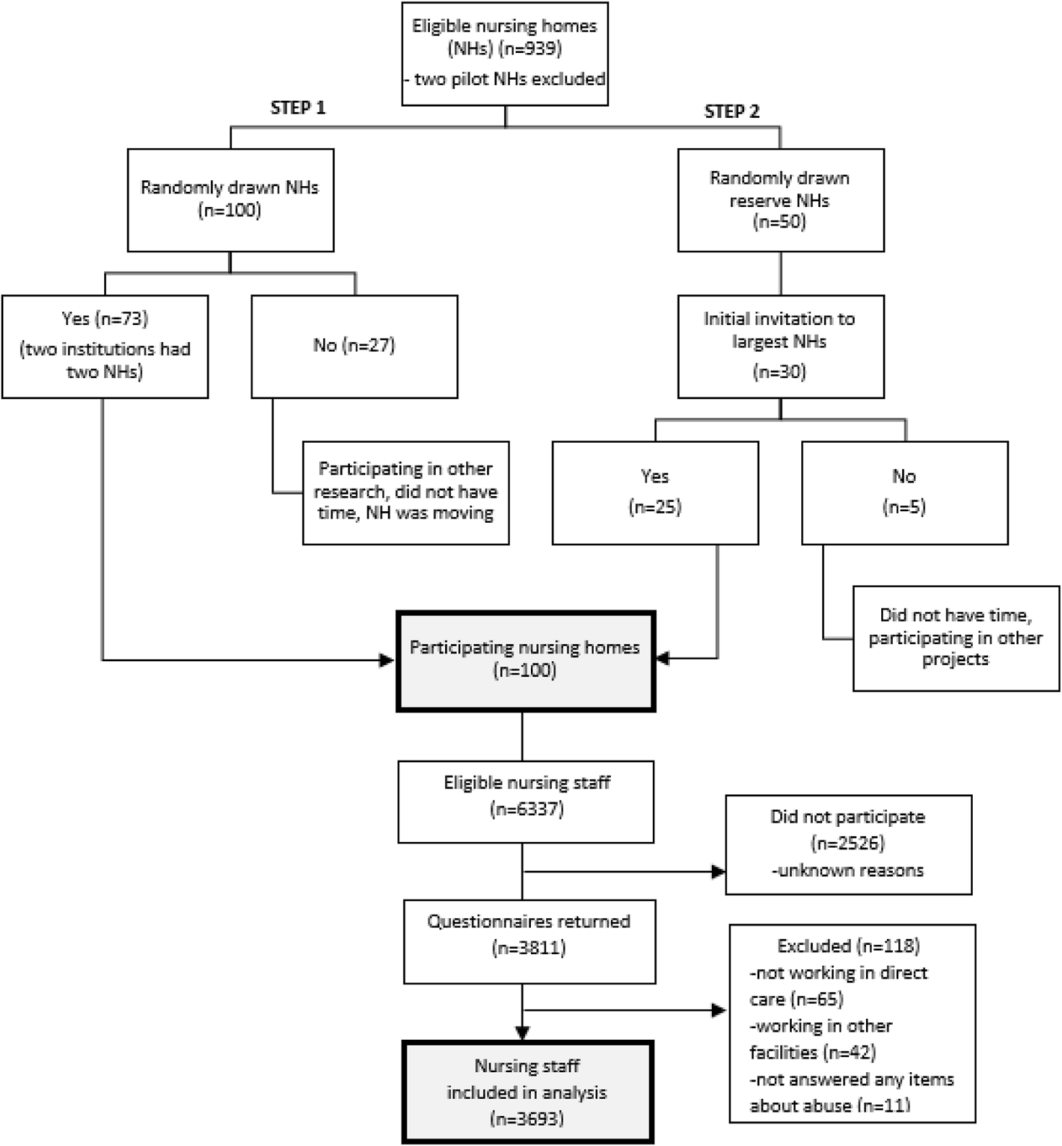
Recruitment of institutions and participants

To obtain a representative sample of institutions (*n* = 939), we used a computerized random number generator to draw a sample of 100 nursing homes, which is approximately 10% of all nursing homes in Norway. All nursing homes had the same statistical chance of being drawn. We also randomly drew 50 nursing homes as reserve homes if institutions declined to participate. Few national studies have been conducted to measure elder abuse in nursing homes, and they all describe different measurement methods. Therefore, we were unable to statistically compute a sample size, but in comparison, the national study in Ireland distributed 3000 questionnaires in 64 nursing homes [24]. To recruit nursing homes, we emailed invitation letters to all nursing home directors, followed by a telephone call. Those who agreed to participate sent a confirmatory email with the potential number of participants at the nursing home and the name of one “coordinator” who could administer the survey. The coordinator task was either assigned to ward managers, the nursing home directors, or others appointed by the directors. Of the 100 invited nursing homes, 27 institutions declined to participate, of which many were above the median size of 34 beds in Norway [44]. To prevent further skewness, we initially invited the 30 largest nursing homes from our reserve list (Fig. 1).

### Participants

Eligible participants were nursing staff; registered nurses, learning disability nurses/social educators, licensed practical nurses, nursing and health care students, and nurse assistants with no formal health education, who worked directly in the care of residents during a three-week period.

Of the nursing staff, 6337 were eligible for inclusion, whereas 3811 returned questionnaires, giving a response rate of 60.1%. Of these, 118 were excluded before analyses because they reported not working in direct care, worked in nursing home day care centres or assisted living facilities, or had not answered any items about abuse. The remaining 3693 nursing staff were included in the statistical analysis, giving an analytic response rate of 58.3% (Fig. 1).

### Variables

The primary outcome measure was to estimate the prevalence of all forms of observed and perpetrated staff-to-resident abuse the past year; psychological, physical, financial/material, sexual and neglect, disaggregated by nursing staff’s gender, age and education.

### Measurements

#### Abuse measurement instrument

To our knowledge, no standardized instrument exists that has been extensively used to measure all types of staff-to-resident abuse as reported by staff in nursing homes. A systematic review by Cooper et al. (2008) [15] reported that one study used a valid and reliable instrument to measure staff-to-resident abuse, but this instrument was limited to measure psychological abuse. Since then, researchers have developed measurement instruments, mainly by adapting items from the widely-used Conflict Tactics Scale (CTS) originally designed to measure intra-family conflict and violence [45]. Few studies have reported psychometric properties of the instruments they have constructed [12]. In our study, we used a questionnaire developed by Dr. Nicholas Castle of the United States, with his permission. The questionnaire has previously been used to measure staff-to-resident and resident-to-resident abuse in four large surveys of staff in US nursing homes and assisted living facilities [21–23, 46]. This questionnaire contained 28 items measuring how often staff observed/perpetrated verbal abuse (5 items), physical abuse (7 items), psychological abuse (3 items), caregiving abuse (2 items), medication abuse (3 items), material exploitation (4 items), and sexual abuse (4 items) towards residents during the past 3 months. The items were scored “Never”, “Once”, “2–3 times”, “4–5 times”, “5–6 times”, and “Other number” and reported with percentages or mean for each questionnaire item. To calculate this mean, positive scoring values (excluding “Never” and “Other number”) were assigned a number from 1 to 4, respectively. The questionnaire demonstrated acceptable internal consistency when measuring observed staff-to-resident abuse in assisted living facilities (Cronbach’s alpha > 0.7) [22].

#### Translation

We used the guidelines for translation and adaptation of instruments previously used by WHO [47]. Initially, two translators forward-translated the instrument from English to Norwegian, and a bilingual expert panel reviewed this and made minor adjustments. We then performed ten cognitive interviews with nursing students working part-time in nursing homes concerning the language and content of the instrument before a professional translator with no knowledge in the field back-translated it to English. The translated version of the instrument was sent to the original author, who had no further comments. To test face validity, the instrument was pretested in a pilot study of 60 nursing staff from two Norwegian nursing homes in June 2018. We also conducted two reflection groups, each with three or four participants, to explore whether the items represented all facets of elder abuse in Norwegian nursing homes.

#### Modification and reliability of instrument

In our study, items of verbal abuse were classified as psychological abuse, and items of medication abuse were classified as physical abuse. We also self-developed and added one item about rape and included six items from the Norwegian study by Malmedal et al. [26] measuring acts of neglect. Overall, our abuse measurement instrument contained 35 items. After the pilot study was carried out, we made some linguistic changes to the questionnaire and added a line detailing that staff should *“not report acts justified in care or treatment i.e. not give food/water to residents before procedures”*. We also altered the scoring values to “Never”, “Once”, “2–5 times”, “6–10 times”, and “More than 10 times”, to measure abuse the past year and not the past 3 months. In our study, the Cronbach’s alpha coefficients were ≥ 0.7 for observed/perpetrated psychological abuse and neglect. We did not conduct a reliability estimation for physical, financial and sexual abuse, because these items/acts represent formative and not reflective measures [48].

#### Final survey questionnaire

The final survey questionnaire contained six sections: (A) participant’s demographic variables (no name or birth date) and employment profile, (B) health status, (C) work-related variables, (D) experiences of conflicts with residents, (E) attitudes towards older people with dementia, and (F) experiences of observed and perpetrated staff-to-resident abuse, observed resident-to-resident abuse and observed relative-to-resident abuse. To gather information about organizational factors i.e. nursing home size and location, number of male/female residents, the nursing home directors and ward managers completed two short questionnaires. In this article, only nursing staff’s gender, age, and education and experiences of observed and perpetrated staff-to-resident abuse are presented.

### Data collection

Packages with instruction letters, survey questionnaires with invitation letter on the first page, and sealed collection boxes were provided to the coordinators at each nursing home. The instruction letter described in detail how the coordinators should administer the survey, and the main author had contact with all coordinators by phone during the data collection period. Participation was voluntary, and no incentive was given directly to participants. We did, however, offer an economic incentive to the eight institutions that achieved the highest response rate, where a sum of approximately 900 GBP was dedicated to the welfare of staff.

### Ethical considerations

All nursing home directors of the randomly drawn nursing homes received information about the study via email and by telephone. Participation was voluntary, and directors who agreed to participate on behalf of the nursing home sent a written consent by email to the main author. Information about the study was given on the first page of the survey questionnaire, and nursing staff participation was voluntary. Since participants did not write their name or birth date on the questionnaire, consent from staff was obtained when they completed and placed the questionnaire in the sealed collection boxes. Staff were informed that they could not withdraw their participation after the questionnaire was returned. All questionnaires were coded so we knew from which nursing home it came, but participants were assured that the code was kept safe by the main author only, and that no participant or nursing home would be identifiable in any publication or report. Due to the nature and sensitivity of the survey questions and the potential of disclosing criminal offences, we applied to the Regional Ethic Committee (REC) for Medical Research. The Committee (REC Central) approved the study in May 2018, reference number: 2018/314.

### Statistical analysis

Data was analysed with Stata 15.2 software package [49]. As in studies with the same scoring values [24, 50], our dependent variable “Abuse” was skewed towards “Never”. For this reason, we dichotomized this variable to “No abuse” (never) and “Abuse” (one or more incidents). Descriptive statistics of nursing staff were presented with frequencies and percentages. Subtypes of abuse were calculated by summarizing all items under the specific category and presented with percentages expressing the number of participants who answered positive (“abuse”) on at least one included item. We did not use a substantive threshold criterion, ten or more incidents during the past year, to define neglect or psychological abuse. Researchers using these criteria report lower prevalence estimates of abuse, and the argument is that one-time scenarios of psychological abuse and neglect cannot be characterized as mistreatment [11]. In the context of nursing homes where the power imbalance is significant as are the vulnerabilities of the residents, we considered one act of abuse to qualify as “Abuse”. Owing to the small rates of financial and sexual abuse, these were not analysed with chi-squared statistics. Nursing staff’s perpetrated acts of psychological abuse, physical abuse and neglect, and nursing staff demographics (gender, age, education) were analysed with Pearson’s Chi-square test. Missing values were removed from all variables. We did not add any design- or post-stratification weights, considering the large sample size.

## Results

Of the 100 participating nursing homes, 48 institutions had ≤34 beds and 52 institutions had > 34 beds, and they ranged in size from eight to 161 beds. Forty-nine nursing homes were in a city, and 94% were publicly run by the municipalities. Of the participants, 63.7% worked in long term care units, 21.8% in dementia special care units, and 14.5% in short-term care units. Most participants were women (91.5%); 37.0% were between 31 and 49 years, and 56.5% had completed high school (Table 1).

**Table 1:** Demographic characteristics of nursing staff (*N* = 3693)

Variables		*n*	%
Gender	Male	312	8.5
	Female	3362	91.5
Age	16–30 years	1000	28.9
	31–49 years	1277	37.0
	50–75 years	1180	34.1
Highest level of education	Primary School	201	5.5
	High School	2050	56.5
	University < 4 years	1126	31.0
	University ≥4 years	253	7.0

Overall, 76% (2435/3204) of nursing staff reported having observed at least one incident of abuse committed by other members of staff, and 60.3% (1881/3124) admitted that they had perpetrated at least one incident of abuse against a resident during the past year.

**Fig. 2:**
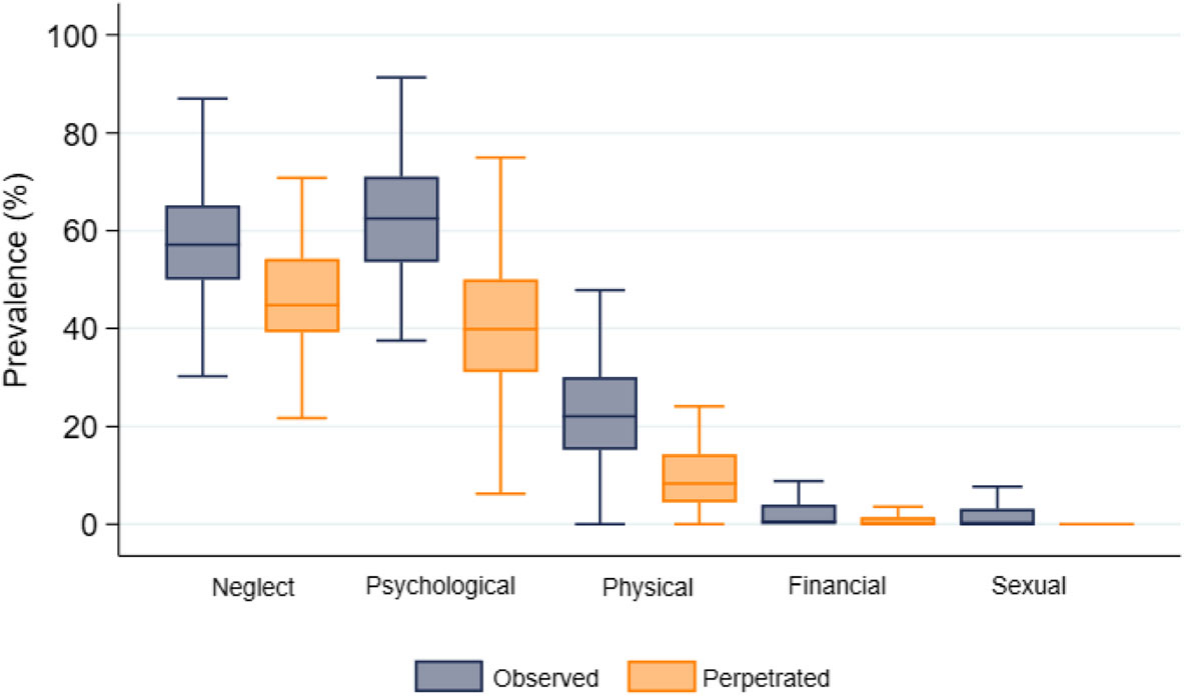
Nursing home (*N* = 100) prevalence rates according to observed/perpetrated elder abuse type

Figure 2 illustrates the prevalence, central tendency and variation in each type of observed and perpetrated abuse in the 100 participating nursing homes, and Table 2 outlines the proportion of each abusive act observed and perpetrated by staff during the past year. Overall, 57.8% (2029/3511) had observed at least one incident of neglect by other staff, with 40.1% (1409/3511) observing staff commit neglectful acts on two or more occasions. The most-frequent reported acts were neglecting oral care (35.4%), ignoring a resident (35.1%), delaying care (29.3%), and prohibiting a resident from using the alarm (20.2%) at least once in the past year.

**Table 2:** Proportion of observed and perpetrated abuse past year, as reported by nursing staff (*N* = 3693)

Type of abuse:		Observed (%):						Perpetrated (%):					
		N	Never	Once	2–5 times	6–10 times	> 10 times	N	Never	Once	2–5 times	6–10 times	> 10 times
Psychological abuse	Yelling at a resident	3621	51.3	15.1	22.0	6.4	5.2	3634	72.9	12.7	11.3	1.8	1.3
	Making nasty remarks to a resident	3609	79.2	8.7	8.4	2.4	1.2	3607	94.1	3.7	1.9	0.2	0.1
	Swearing at a resident	3632	88.2	5.3	4.8	0.9	0.8	3638	95.1	2.9	1.6	0.1	0.3
	Making humiliating remarks to a resident	3590	81.5	7.9	7.8	1.7	1.2	3593	94.5	3.1	1.5	0.5	0.5
	Arguing with a resident	3611	63.2	14.2	16.1	3.7	2.8	3618	78.6	11.3	8.2	1.1	0.9
	Making threatening remarks to a resident	3615	93.3	3.6	2.2	0.5	0.4	3624	97.9	1.4	0.5	0.1	0.06
	Making critical remarks to a resident	3615	78.2	9.3	9.7	1.7	1.2	3622	90.2	6.0	3.3	0.2	0.3
	Threatening to stop taking care of a resident	3643	87.9	5.0	5.7	0.7	0.7	3624	94.3	3.2	1.9	0.4	0.3
Physical abuse	Pushing, grabbing, or pinching a resident	3599	87.1	6.4	4.7	0.9	0.9	3606	94.2	3.5	1.6	0.3	0.5
	Pulling hair or kicking a resident	3608	99.2	0.5	0.3	0.06	0.03	3620	99.7	0.1	0.1	0.03	0.03
	Hurting a resident on purpose	3611	99.4	0.4	0.2	–	0.03	3625	99.9	0.08	–	–	0.03
	Throwing things at a resident	3611	99.3	0.4	0.3	–	0.06	3616	99.9	0.06	0.03	–	0.03
	Hitting a resident	3611	99.2	0.4	0.3	0.03	0.06	3622	99.9	0.06	0.03	–	0.03
	Bullying a resident	3606	96.3	2.2	1.1	0.2	0.2	3616	99.6	0.3	0.03	–	0.03
	Behaving aggressively towards a resident	3610	91.6	4.3	3.1	0.6	0.4	3606	98.0	1.3	0.6	0.06	0.06
	Not giving needed medication on purpose to a resident	3640	98.6	0.7	0.4	0.1	0.1	3629	99.7	0.08	0.1	0.03	0.06
	Giving more medication than needed on purpose to a resident	3636	96.2	1.8	1.4	0.4	0.3	3630	99.2	0.4	0.3	0.1	0.06
	Deliberately delaying giving medication(s) to a resident	3626	95.5	1.4	2.2	0.5	0.4	3619	97.2	1.0	1.2	0.3	0.4
Financial/material abuse	Stealing money from a resident	3615	99.6	0.3	0.1	0.03	–	3626	99.9	0.03	0.03	–	0.03
	Stealing things from a resident	3619	99.5	0.3	0.1	0.08	0.03	3625	99.9	0.06	0.03	–	0.03
	Signing documents without permission from a resident	3617	99.3	0.5	0.1	–	0.06	3630	99.7	0.3	0.03	–	–
	Destroying things that belong to a resident without permission	3616	99.2	0.5	0.3	–	0.06	3631	99.3	0.6	0.08	–	–
Sexual abuse	Unwelcome touching of a resident	3617	99.7	0.2	0.03	0.03	0.03	3627	99.9	0.08	0.06	–	–
	Unwelcome discussion of sexual activity with a resident	3615	98.9	0.8	0.3	0.03	0.03	3624	99.7	0.2	0.03	–	0.06
	Exposure of a residents private-body parts to embarrass them	3610	99.7	0.08	0.08	0.08	0.03	3624	100	–	–	–	–
	Digital penetration (e.g. finger) of a resident	3613	100	–	–	–	–	3622	100	–	–	–	–
	Rape of a resident	3614	100	–	–	–	–	3618	100	–	–	–	–
Neglect	Not giving food on purpose to a resident	3638	96.7	1.8	1.1	0.2	0.2	3634	99.2	0.4	0.2	0.08	0.06
	Not giving fluid on purpose to a resident	3646	97.4	1.2	1.1	0.1	0.2	3642	99.3	0.4	0.2	–	0.1
	Delaying care of a resident	3643	70.7	7.6	14.7	3.0	3.9	3622	80.5	6.9	8.8	1.5	2.4
	Ignoring a resident	3626	64.9	8.3	17.6	4.4	4.9	3613	74.7	8.3	11.9	2.3	3.0
	Not treating a resident’s wounds carefully enough	3628	90.1	3.8	4.7	0.9	0.5	3611	95.9	2.7	1.2	0.1	0.03
	Neglecting oral care of a resident	3608	64.6	6.0	17.2	5.8	6.5	3589	69.5	8.8	15.4	3.2	3.3
	Not changing diapers on a resident	3627	81.1	5.3	8.5	2.6	2.5	3626	89.8	5.0	3.9	0.6	0.7
	Prohibiting a resident from using the alarm	3638	79.8	6.2	10.1	1.8	2.2	3633	88.3	5.1	4.8	0.6	1.3

Overall, 62.4% (2155/3452) had observed at least one incident of psychological abuse committed by other staff in the past year, with 43.4% (1499/3452) reporting they had observed such abusive acts on two or more occasions. Incidents of yelling were most prevalent with almost 50% of staff observing this at least once, followed by arguing with a resident (36.8%) and making critical remarks to a resident (21.8%) at least once during the past year. Regarding physical abuse, 23.2% (810/3489) had observed staff commit one or more acts, and 8.7% (305/3489) had observed this on two or more occasions. The most frequent acts were pushing, grabbing or pinching a resident (12.9%), behaving aggressively towards a resident (8.4%), and deliberately delaying giving medications (4.5%) at least once in the past year. Most nursing staff reported that they had never observed financial/material abuse (97.9%, 3514/3591) or sexual abuse of residents (98.4%, 3525/3583).

Overall, 46.9% (1623/3460) of staff admitted perpetrating at least one incident of neglect in the past year, and 27.6% (954/3460) had done this on two or more occasions. Like observed abuse, the most frequent act was neglecting oral care (30.5%), ignoring residents when they called (25.3%), deliberately delaying care (19.5%), and prohibiting residents from using the alarm (11.7%).Overall, 40.5% (1387/3427) admitted they had perpetrated at least one act of psychological abuse, with 21.5% (737/3427) admitting they had done this on two or more occasions. Like observed abuse, most staff admitted yelling at a resident (27.1%) and arguing with a resident (21.4%). Regarding physical abuse, 9.6% (335/3477) admitted perpetrating these acts at least once, and 2.2% (76/3477) had done this at least twice. Regarding physical acts, 5.8% of staff admitted pushing, grabbing or pinching a resident, and 4.5% had deliberately delayed giving a resident medication. The majority of staff reported they had never committed financial/material abuse (98.9%, 3559/3600), or sexual abuse against a resident (99.6%, 3565/3578).

Table 3 outlines nursing staff characteristics associated with self-reported perpetrated abuse. A significantly higher proportion of males reported committing physical abuse, and a higher proportion of females admitted acts of neglect. We found no significant differences between age groups and abuse. Higher-educated staff admitted more acts of psychological abuse, physical abuse, and neglect.

**Table 3:** Nursing staff demographics and self-reported perpetrated psychological abuse, physical abuse and neglect

	Psychological, % (n)			Physical, % (n)			Neglect, % (n)		
Staff characteristics	No Abuse	Abuse	*p*-value*	No Abuse	Abuse	*p*-value*	No Neglect	Neglect	*p*-value*
Gender									
Male	57.4 (163)	42.6 (121)	0.437	84.9 (248)	15.1 (44)	**0.001**	59.4 (171)	40.6 (117)	**0.026**
Female	59.8 (1868)	40.2 (1258)		90.8 (2880)	9.2 (291)		52.5 (1657)	47.5 (1498)	
Age									
16–30 years	58.7 (550)	41.3 (387)	0.791	90.6 (858)	9.4 (89)	0.706	55.0 (518)	45.0 (424)	0.244
31–49 years	58.9 (695)	41.1 (486)		89.8 (1080)	10.2 (123)		51.5 (615)	48.5 (579)	
50–75 years	60.0 (671)	40.0 (447)		90.7 (1026)	9.3 (105)		52.1 (583)	47.9 (536)	
Education									
Primary school	72.6 (130)	27.4 (49)	**0.001**	96.1 (174)	3.9 (7)	**0.003**	68.4 (121)	31.6 (56)	**0.000**
High School	59.8 (1142)	40.2 (769)		91.0 (1762)	9.0 (174)		54.0 (1039)	46.0 (886)	
University < 4 years	57.3 (603)	42.7 (450)		88.1 (946)	11.9 (128)		48.9 (519)	51.1 (543)	
University ≥4 years	54.9 (129)	45.1 (106)		89.6 (215)	10.4 (25)		50.8 (123)	49.2 (119)	

^*^Pearson’s Chi-square test

## Discussion

Our findings demonstrate that approximately two-thirds of staff in Norwegian nursing homes reported having committed one or more acts of resident mistreatment during the past year, with neglect and psychological abuse being the most commonly reported. The overall prevalence rate of perpetrated staff-to-resident abuse in our study is slightly lower than the pooled estimate reported in the meta-analysis of Yon et al. (2019), but we found a slightly higher prevalence rate of psychological abuse and a considerably higher rate of neglect than the meta-analysis. Furthermore, when we compared our results to the national study of staff-to-resident abuse in Ireland, we found significantly higher rates of all types of abuse except *observed* neglect [24]. These differences could be explained by the fact that we used other operational definitions of abuse than the Irish study, and we also used more items in each subcategory to measure the mistreatment of residents.

Prevalence rates of perpetrated abuse were lower than rates of observed abuse, which is consistent with findings in other studies [24, 27]. This might indicate that staff find it easier to report abuse they observe committed by colleagues rather than admitting their own abusive behaviour. Moreover, we found a smaller difference between observed and perpetrated neglect than the other subtypes of abuse, and a possible explanation might be that staff perceive neglect as systemic failures rather than their personal responsibilities and therefore easier to admit [27]. For example, neglecting oral care was the most frequently reported act of neglect in our study and in the Norwegian study from 2009 [26]. Neglecting oral care may be due to factors such as lack of time or adequate equipment, inadequate training/experience in delivering oral care, or residents’ resistance to care [51, 52] rather than due to negative motivations. Still, intentional or unintentional, personal or systemic failure; adequate oral hygiene is crucial for a person’s general health and well-being [53].

Psychological abuse is reported as the most prevalent type of abuse in many studies [21, 25, 27–29], and we also found a high prevalence rate of both observed and perpetrated psychological abuse. The most frequently reported act in our study was staff yelling at a resident, which is consistent with prevalence rates reported by nursing home staff in the Czech Republic [29] and by nurses in German nursing homes [27], but quite in contrast to the low rate found in Irish nursing homes [24]. One might argue that “yelling” or “arguing” with residents are not abusive acts but basic features in the daily life of a nursing home [33], which might be supported by a study that found that staff used verbal aggression to keep control and “order” within the institution, thereby normalizing and neutralizing such acts [27]. As our findings came from nurses’ interpretations of “yelling” and “arguing” these terms should be clarified in qualitative interviews with staff in order to further interpret this finding. Nevertheless, in the context of a nursing home, healthcare professionals are in a position of power and control over vulnerable adults, and acts of verbal aggression are considered intimidating and disrespectful [50].

Older adults are more vulnerable and physically weaker than younger people, and even minor physical injuries can create serious or long-lasting damage [34]. We found that approximately 10% of nursing staff admitted perpetrating acts of physical character, which is in line with rates in the Czech Republic [29], but lower than rates in German nursing homes [27]. Again, our prevalence rate of physical abuse was higher than in Irish nursing homes [24]. According to the CDC’s definition, acts of medication abuse are considered physical abuse [9], this in contrast to the definitions used in the Irish study where medication abuse was considered neglect [24]. Hence, we found that a very small proportion of staff admitted perpetrating medication abuse in our study.

The prevalence of both observed and perpetrated financial/material abuse was low in our study, still slightly higher than the Irish study [24], but lower than the rates reported in US and Croatian nursing homes [28, 31]. There are 30 or more ways older people can be financially exploited [16], and we only used four items which might explain of our low estimate. Nevertheless, Neuberg et al. (2017) [28] used a single item to measure financial abuse in Croatian nursing homes and reported a higher prevalence rate than all these mentioned studies. In 2018, about 100 Norwegian health care providers lost their licenses due to substance abuse or drug theft [54], and retrospectively, we should have added a question concerning staff stealing drugs from residents.

The prevalence rate of sexual abuse was low in our study, which is consistent with other studies [21, 24, 29]. Sexual assault is one of the most shocking types of abuse, and therefore considered the most hidden and least acknowledged [17]. Ageism and negative stereotypes towards older adults’ sexuality might impede nursing staff in recognizing sexual abuse of residents, thus staff need better knowledge and training in the detection, examination and managing of sexual assaults in nursing homes [55].

To examine individual-level risk factors of abuse (ecological micro-level), we disaggregated the subtypes according to nursing staff demographics and found that certain individual appearances were associated with higher rates of abuse. One interesting finding was that more women than men admitted acts of neglect. To our knowledge, this is not reported elsewhere, and it is inconsistent with the Irish study where men reported higher rates of neglect [24]. Stress and caregiver burnout is found to be associated with elder abuse [37], and a plausible explanation might be that more women in our sample suffered from burnout. A meta-analysis of gender differences in burnout did find that women were slightly more emotionally exhausted than men, but they also found that men were more depersonalized [56]. Concerning physical abuse, we found that more men than women reported acts of physical character, which is consistent with the finding in Swedish nursing homes [32]. Men might be allocated to work with certain set of tasks e.g. people who are challenging or agitated, hence conduct and report more physical behaviours than women [57]. Nevertheless, these gender differences are not easily explained, and they should be further explored.

Educated staff in our study reported more incidents of all types of abuse, and this was also found in Norwegian nursing homes in 2009 [26]. Nursing staffs’ technical expertise, experience, and ability to critical thinking influence quality of health care [58]. In Norway, nurse aides are certified health practitioners after finishing high school, and we speculate whether health educated staff reflect more critically upon their practice and therefore recognize and self-report more acts of abuse compared to the non-certified nurse assistants.

Detecting the extent of elder abuse is inherently difficult, and our study has certain limitations. Firstly, even though the nursing homes were randomly drawn, some institutions declined to participate, and more of the larger nursing homes rejected participation in the initial recruitment phase. These nursing homes did not differ from the rest of the sample with respect to how they were run or located, but one could speculate whether more “problematic” institutions were less likely to accept our invitation. Secondly, our study was based on self-reports by staff, which might have caused response bias, such as social desirability not to report sensitive/incriminating acts of abuse and recall bias when they were asked to remember the exact number observed/perpetrated incidents during the past year. We are also uncertain how staff interpreted the instruction of *“do not report acts justified in health care or treatment”*, where they could have failed to interpret their own misconduct as abusive. We found higher prevalence rates when staff reported on colleagues’ behaviours than what they admitted themselves, which could be an indicator of underreporting, but also the result of several staff observing the same incidents of abuse. Thirdly, we did not test the formative measurements of sexual, financial/material and physical abuse, which should take place in future studies. Finally, the cross-sectional study design offers no information about causal relationships between risk factors and abuse.

A strength of our study was the large sample size of 100 nursing homes and 3693 staff, which makes it one of the largest studies exploring the prevalence of staff-to-resident abuse in institutional settings. We also achieved a relatively high response rate of 60.1% compared to other elder abuse studies with response rates ranging from 22 to 43% [24, 27, 31]. These strengths allow us to generalize our results to the rest of the Norwegian nursing home population.

The findings in our study may have practical and theoretical implications for policy, research, care and education. Firstly, nearly all US-states and some countries in the European region have mandatory reporting legislation that requires healthcare staff to report suspicions of elder mistreatment [37, 59]. In Norway, explicit laws against child maltreatment, intimate partner violence and sexual violence exist, but no specific laws against elder abuse [10]. Nevertheless, according to the recent amendment (2017) in the Norwegian Health and Care Services Act, nursing staff have a professional responsibility to detect and prevent violence and sexual abuse against all patients in municipal health and care services [60]. The risks and benefits of mandatory reporting deserve more study so that the laws may be written in such a way as to minimize harm and maximize value.

In England and Wales, social care staff are legally required to report employees committing misconduct of vulnerable adults, to the Protection of Vulnerable Adults (POVA) list, which may ban employees from similar employment [57]. A list or register like this should be studied and considered in all countries. Adult Protective Services (APS) is a social services model adopted by the US designed to investigate mistreatment of vulnerable adults, but only 34% of countries in the world have applied such a model [10]. Norway has child protective services but no adult protective services, and according to the high prevalence rates in our study and in the Norwegian study of Sandmoe et al. (2017) [39], Norwegian policy-makers should consider establishing services that also protect and serve vulnerable adults exposed to mistreatment. The APS model has not been rigorously studied but may serve as a model that may be adapted to fit the needs of other countries.

To understand why prevalence rates of staff-to-resident abuse are so alarmingly high, we need more research on the underlying risk factors within all levels of the ecological framework. Moreover, nursing staff are in a unique position to detect elder mistreatment, and we need to develop, implement and evaluate interventions to make staff better equipped to observe, handle and report incidents of suspected/alleged abuse, but also interventions that prevent health professionals from committing acts of abuse. Public awareness campaigns and educational programmes for healthcare staff are vital interventions to reduce and prevent elder abuse, and this can be conducted in a variety of ways including training courses, workshops, educational seminars, scientific meetings and conferences [34]. Several interventions have been implemented to reduce the occurrence of elder abuse in both community and institutional settings, but there is still ambiguity whether these interventions improve knowledge and attitude of caregivers, and future studies are warranted [61].

## Conclusions

This is the first national study to examine the prevalence of staff-to-resident abuse in Norwegian nursing homes, and it is one of the largest studies to estimate the prevalence of elder abuse in institutional settings worldwide. Our findings demonstrate that resident abuse is a relatively common problem in Norwegian nursing homes, and residents are exposed to many forms of mistreatment.

We believe our study provides significant knowledge about the extent and nature of staff-to-resident abuse in institutional care settings, and our findings are important for Norwegian policy makers when developing future strategic white papers and national action plans to address and prevent elder abuse. Furthermore, our large survey of staff provides essential information about resident abuse in institutional care that future national and international researchers might use to plan and implement measures that could improve the quality of life and safety of older people.

## Data Availability

The dataset generated and analyzed during the current study are available from the corresponding author on reasonable request.

## Abbreviations

APS: Adult Protective Services
CDC: Centers for Disease Control and Prevention
CTS: Conflict Tactics Scale
NH: Nursing home
NTNU: Norwegian University of Science and Technology
POVA: Protection of Vulnerable Adults
REC: Regional Ethics Committee
US: United States
WHO: World Health Organization

## References

[1] Schofield MJ, Powers JR, Loxton D (2013). Mortality and disability outcomes of self-reported elder abuse: a 12-year prospective investigation. J Am Geriatr Soc.

[2] Olofsson N, Lindqvist K, Danielsson I (2012). Fear of crime and psychological and physical abuse associated with ill health in a Swedish population aged 65-84 years. Public Health.

[3] Baker MW, LaCroix AZ, Wu C, Cochrane BB, Wallace R, Woods NF (2009). Mortality risk associated with physical and verbal abuse in women aged 50 to 79. J Am Geriatr Soc.

[4] Dong X, Simon MA (2013). Elder abuse as a risk factor for hospitalization in older persons. JAMA Intern Med.

[5] Dong X, Simon MA (2013). Association between reported elder abuse and rates of admission to skilled nursing facilities: findings from a longitudinal population-based cohort study. Gerontology..

[6] Yunus RM, Hairi NN, Choo WY (2019). Consequences of elder abuse and neglect: a systematic review of observational studies. Trauma Violence Abuse.

[7] Dong X, Simon MA (2013). Association between elder abuse and use of ED: findings from the Chicago health and aging project. Am J Emerg Med.

[8] Butchart A, Brown D, Khanh-Huynh A, Corso P, Florquin N, Muggah R (2008). Manual for estimating the economic costs of injuries due to interpersonal and self-directed violence.

[9] Hall JE, Karch DL, Crosby AE (2016). Elder abuse surveillance: uniform definitions and recommended Core data elements for use in Elde abuse surveillance, version 1.0.

[10] World Health Organization (2014). Global status report on violence prevention 2014.

[11] Pillemer K, Burnes D, Riffin C, Lachs MS (2016). Elder abuse: global situation, risk factors, and prevention strategies. Gerontologist.

[12] Ho CS, Wong SY, Chiu MM, Ho RC (2017). Global prevalence of elder abuse: a meta-analysis and meta-regression. East Asian Arch Psychiatr.

[13] McDonald L, Beaulieu M, Harbison J, Hirst S, Lowenstein A, Podnieks E (2012). Institutional abuse of older adults: what we know, what we need to know. J Elder Abuse Negl.

[14] Yon Y, Ramiro-Gonzalez M, Mikton CR, Huber M, Sethi D (2019). The prevalence of elder abuse in institutional settings: a systematic review and meta-analysis. Eur J Pub Health.

[15] Cooper C, Selwood A, Livingston G (2008). The prevalence of elder abuse and neglect: a systematic review. Age Ageing.

[16] Jackson SL (2018). A systematic review of financial exploitation measures in prevalence studies. J Appl Gerontol.

[17] Smith D, Bugeja L, Cunningham N, Ibrahim JE (2018). A systematic review of sexual assaults in nursing homes. Gerontologist.

[18] Yon Y, Mikton CR, Gassoumis ZD, Wilber KH (2017). Elder abuse prevalence in community settings: a systematic review and meta-analysis. Lancet Glob Health.

[19] Dong X, Chen R, Chang ES, Simon M (2013). Elder abuse and psychological well-being: a systematic review and implications for research and policy--a mini review. Gerontology.

[20] Castle N, Ferguson-Rome JC, Teresi JA (2015). Elder abuse in residential long-term care: an update to the 2003 National Research Council report. J Appl Gerontol.

[21] Castle N (2012). Nurse Aides' reports of resident abuse in nursing homes. J Appl Gerontol.

[22] Castle N (2013). An examination of resident abuse in assisted living Facilites.

[23] Castle N, Beach S (2013). Elder abuse in assisted living. J Appl Gerontol.

[24] Drennan J, Lafferty A, Treacy P, Fealy G, Phelan A, Lyons I, et al. Older people in residential care settings: results of a National Survey of staff-resident interactions and conflicts: University College Dublin: National Centre for the Protection of Older People; 2012.

[25] Blumenfeld Arens O, Fierz K, Zuniga F (2017). Elder abuse in nursing homes: do special care units make a difference? A secondary data analysis of the Swiss nursing homes human resources project. Gerontology.

[26] Malmedal W, Ingebrigtsen O, Saveman BI (2009). Inadequate care in Norwegian nursing homes - as reported by nursing staff. Scand J Caring Sci.

[27] Goergen T (2004). A multi-method study on elder abuse and neglect in nursing homes. J Adult Prot.

[28] Neuberg M, Zeleznik D, Mestrovic T, Ribic R, Kozina G (2017). Is the burnout syndrome associated with elder mistreatment in nursing homes: results of a cross-sectional study among nurses. Arh Hig Rada Toksikol.

[29] Buzgova R, Ivanova K (2011). Violation of ethical principles in institutional care for older people. Nurs Ethics.

[30] Natan MB, Lowenstein A, Eisikovits Z (2010). Psycho-social factors affecting elders' maltreatment in long-term care facilities. Int Nurs Rev.

[31] Harris DK, Benson ML (1999). Theft in nursing homes: an overlooked form of elder abuse. J Elder Abuse Negl.

[32] Saveman BI, Astrom S, Bucht G, Norberg A (1999). Elder abuse in residential settings in Sweden. J Elder Abuse Negl.

[33] Pillemer K, Moore DW (1989). Abuse of patients in nursing-homes - findings from a survey of staff. Gerontologist.

[34] Krug EG, Dahlberg LL, Mercy J, Zwi AB, Lozano R (2002). World report on violence and health.

[35] Schiamberg LB, Gans D (2000). Elder abuse by adult children: an applied ecological framework for understanding contextual risk factors and the intergenerational character of quality of life. Int J Aging Hum Dev.

[36] Schiamberg LB, Barboza GG, Oehmke J, Zhang Z, Griffore RJ, Weatherill RP (2011). Elder abuse in nursing homes: an ecological perspective. J Elder Abuse Negl.

[37] Sethi D, Wood S, Mitis F, Bellis M, Penhale B, Marmolejo II (2011). European report on preventing elder maltreatment.

[38] Wang JJ (2005). Psychological abuse behavior exhibited by caregivers in the care of the elderly and correlated factors in long-term care facilities in Taiwan. J Nurs Res.

[39] Sandmoe AW-LT, Hjemdal OK (2017). Violence and Abuse against Elderly People in Norway.

[40] Syse A, Pham DQ, Keilman N (2016). Befolkningsframskrivinger 2016–2100: Dødelighet og levealder.

[41] Sykepleien. [SSB: Norge vil mangle 28 000 sykepleiere i 2035] [Internet]. Oslo: Sykepleien; 2019 [updated 09.05.2019; cited 2019 29.06]. Available from: https://sykepleien.no/2019/05/ssb-norge-vil-mangle-28-000-sykepleiere-i-2035.

[42] Gautun HØH, Bratt C (2016). Underbemanning er selvforsterkende. Konsekvenser av mangel på sykepleiere i hjemmesykepleien og sykehjem.

[43] Ringard A, Sagan A, Sperre Saunes I, Lindahl AK (2013). Norway: health system review. Health Syst Transit.

[44] Norway S (2017). KOSTRA omsorgsstatistikk 2017, upublisert tall.

[45] Straus MA (1979). Measuring intra-family conflict and violence - conflict tactics (Ct) scales. J Marriage Fam.

[46] Castle NG (2012). Resident-to-resident abuse in nursing homes as reported by nurse aides. J Elder Abuse Negl.

[47] World Health Organization. Process of translation and adaptation on instruments [Internet]: World Health Organization. Available from: http://www.who.int/substance_abuse/research_tools/translation/en/, [cited 2019 29.06]

[48] Edwards JR, Bagozzi RP (2000). On the nature and direction of relationships between constructs and measures. Psychol Methods.

[49] StataCorp. Stata Statistical Software: Release 15. College Station, TX: StataCorp LLC2017.

[50] Pillemer K, Bachmanprehn R (1991). Helping and hurting - predictors of maltreatment of patients in nursing-homes. Res Aging.

[51] Willumsen T, Karlsen L, Naess R, Bjorntvedt S (2012). Are the barriers to good oral hygiene in nursing homes within the nurses or the patients?. Gerodontology.

[52] Bots-VantSpijker PC, Bruers JJ, Bots CP, Vanobbergen JN, De Visschere LM, de Baat C (2016). Opinions of dentists on the barriers in providing oral health care to community-dwelling frail older people: a questionnaire survey. Gerodontology.

[53] Astvaldsdottir A, Bostrom AM, Davidson T, Gabre P, Gahnberg L, Sandborgh Englund G (2018). Oral health and dental care of older persons-a systematic map of systematic reviews. Gerodontology.

[54] The Norwegian Board of Health Supervision. [Reaksjoner mot helsepersonell og virksomheter i helse- og omsorgstjenesten i 2018] [Internet]. Oslo: The Norwegian Board of Health Supervision; 2019 [updated 04.02.2019; cited 2019 29.06]. Available from: https://www.helsetilsynet.no/presse/nyhetsarkiv/2019/reaksjoner-mot-helsepersonell-og-virksomheter-i-helse%2D%2Dog-omsorgstjenesten-i-2018/.

[55] Connolly MT, Breckman R, Callahan J, Lachs M, Ramsey-Klawsnik H, Solomon J (2012). The sexual Revolution's last frontier: how silence about sex undermines health, well-being, and safety in old age. Generations.

[56] Purvanova RK, Muros JP (2010). Gender differences in burnout: a meta-analysis. J Vocat Behav.

[57] Hussein S, Stevens M, Manthorpe J, Rapaport J, Martineau S, Harris J (2009). Banned from working in social care: a secondary analysis of staff characteristics and reasons for their referrals to the POVA list in England and Wales. Health Soc Care Community.

[58] Benner P, Hughes RG, Sutphen M. Clinical Reasoning, Decisionmaking, and Action: Thinking Critically and Clinically. In: Hughes RG, editor. Patient Safety and Quality: An Evidence-Based Handbook for Nurses. Advances in Patient Safety. Rockville (MD)2008. 21328745

[59] Dong XQ (2015). Elder abuse: systematic review and implications for practice. J Am Geriatr Soc.

[60] The Norwegian Health and Care Services Act. In: Ministry of Health and Care Services, editor. 2011.

[61] Baker PR, Francis DP, Mohd Hairi NN, Othman S, Choo WY (2017). Interventions for preventing elder abuse: applying findings of a new Cochrane review. Age Ageing.

